# Prey-dependent retention of dimethylsulfoniopropionate (DMSP) by mixotrophic dinoflagellates

**DOI:** 10.1111/j.1462-2920.2011.02600.x

**Published:** 2012-03

**Authors:** Hyunwoo Lee, Ki-Tae Park, Kitack Lee, Hae Jin Jeong, Yeong Du Yoo

**Affiliations:** 1 School of Environmental Science and Engineering, Pohang University of Science and TechnologyPohang 790-784, Korea; 2 School of Earth and Environmental Sciences, Seoul National UniversitySeoul 151-747, Korea

## Abstract

We investigated the retention of dimethylsulfoniopropionate (DMSP) in phototrophic dinoflagellates arising from mixotrophy by estimating the cellular content of DMSP in *Karlodinium veneficum* (mixotrophic growth) fed for 7–10 days on either DMSP-rich *Amphidinium carterae* (phototrophic growth only) or DMSP-poor *Teleaulax* sp. (phototrophic growth only). In *K. veneficum* fed on DMSP-poor prey, the cellular content of DMSP remained almost unchanged regardless of the rate of feeding, whereas the cellular content of DMSP in cells of *K. veneficum* fed on DMSP-rich prey increased by as much as 21 times the cellular concentration derived exclusively from phototrophic growth. In both cases, significant fractions (10–32% in the former case and 55–65% in the latter) of the total DMSP ingested by *K. veneficum* were transformed into dimethylsulfide and other biochemical compounds. The results may indicate that the DMSP content of prey species affects temporal variations in the cellular DMSP content of mixotrophic dinoflagellates, and that mixotrophic dinoflagellates produce DMS through grazing on DMSP-rich preys. Additional studies should be performed to examine the universality of our finding in other mixotrophic dinoflagellates feeding on diverse prey species.

## Introduction

Since the Gaia hypothesis was proposed as a possible regulatory mechanism for the earth's climate, involving alterations to the global radiation balance (Lovelock *et al*., 1972; Charlson *et al*., 1987), numerous studies have investigated the formation of dimethylsulfide (DMS) in marine environments and its ultimate release to the atmosphere. DMS is ubiquitous at less than nanomolar concentrations in surface waters. The total DMS emitted to the atmosphere from the ocean surface each year makes a major contribution to the atmospheric sulfur budget (Kettle and Andreae, 2000). The main precursor of DMS is dimethylsulfoniopropionate (DMSP) (Stefels *et al*., 2007), a compatible solute synthesized by various groups of marine algae, among which dinoflagellates and prymnesiophytes contain relatively high concentrations (Keller *et al*., 1989). DMSP may have multiple functions including as an osmolyte, a cryoprotectant (Kirst *et al*., 1991), an antioxidant (Sunda *et al*., 2002), a predatory deterrent (Storm *et al*., 2003) and a chemo-attraction (Seymour *et al*., 2010). The transformation of DMSP into DMS is controlled by several processes that have yet to be fully elucidated (Stefels *et al*., 2007). The enzyme DMSP lyase is known to be involved in DMS production processes including direct release from phytoplankton, viral infection and grazing and bacterial activity (e.g. Wolfe and Steinke, 1996; Malin *et al*., 1998; Zubkov *et al*., 2001). Other identified pathways of DMSP are its cleavage into DMS by hydroxide ions (Dacey and Blough, 1987) and bacterial gene *dddD* (Todd *et al*., 2007).

Phototrophic dinoflagellates were initially thought to be exclusively autotrophic, but many are now known to be mixotrophic (i.e. capable of both photosynthesis and ingestion of prey) (e.g. Jacobson and Anderson, 1996; Stoecker, 1999; Jeong *et al*., 2004). They are also major producers of DMSP among the phytoplankton groups, although their cellular DMSP content is highly variable (Keller *et al*., 1989). Some species express DMSP lyase, and consequently form DMS during algal blooms (Steinke *et al*., 2002). Recent studies have shown that significant quantities of ingested DMSP are used by heterotrophic dinoflagellates as a source of intracellular sulfur-containing compounds (Tang and Simó, 2003; Saló*et al*., 2009). This finding is probably also applicable to mixotrophic dinoflagellates when they feed on prey. During grazing, the breakdown of DMSP-containing cells mixes the intracellular DMSP and DMSP-lyase enzymes, whichare otherwise physically separated inside the cell, enabling the enzyme catalysed transformation of cellular DMSP to DMS (Wolfe and Steinke, 1996).

The unique characteristics of mixotrophic dinoflagellates described above indicate that they are potentially important in the dynamics of oceanic DMSP and DMS. However, our knowledge of the roles of dinoflagellates in the formation of DMSP and DMS is far less than that for other DMS producing species (e.g. *Emiliania huxleyi* and *Phaeocystis* spp.), which have been the focus of previous studies (Matrai and Keller, 1993; Burkill *et al*., 2002). We report here that the cellular content of DMSP in a mixotrophic dinoflagellate varied with the DMSP content of the ingested prey species. We established two sets of experimental bottles, one containing a mixture of the mixotrophic dinoflagellate predator *Karlodinium veneficum* and its mixotrophic dinoflagellate prey *Amphidinium carterae* (DMSP-rich), and the other containing a mixture of *K. veneficum* and the cryptophyte prey *Teleaulax* sp. (DMSP-poor). We also established controls containing *K. veneficum*, *A. carterae* and *Teleaulax* sp. alone. We measured the rate of ingestion of *A. carterae* and *Teleaulax* sp. by *K. veneficum* in the experimental bottles, and the DMSP concentration in the experimental and control (predator and prey alone) bottles. The results of the study provide insights into the possible involvement of the mixotrophic nature of dinoflagellates in the ocean sulfur cycle.

## Results and discussion

### Mixotrophic nature of *K. veneficum*

The mixotrophic nature of *K. veneficum* feeding on a cryptophyte species (e.g. *Storeatula major*) was identified in previous studies (e.g. Li *et al*., 1996; 1999; Adolf *et al*., 2006; 2008). Our study provides the first evidence that *K. veneficum* also feeds on the dinoflagellate species *A. carterae.* The transmission electron micrograph shows a cell of *A. carterae* inside a *K. veneficum* cell (Fig. S1). The predator *K. veneficum* is known to use toxins to capture prey cells (Sheng *et al*., 2010) and feeds on them by phagocytic engulfment (Li *et al*., 2000).

**Fig 1:**
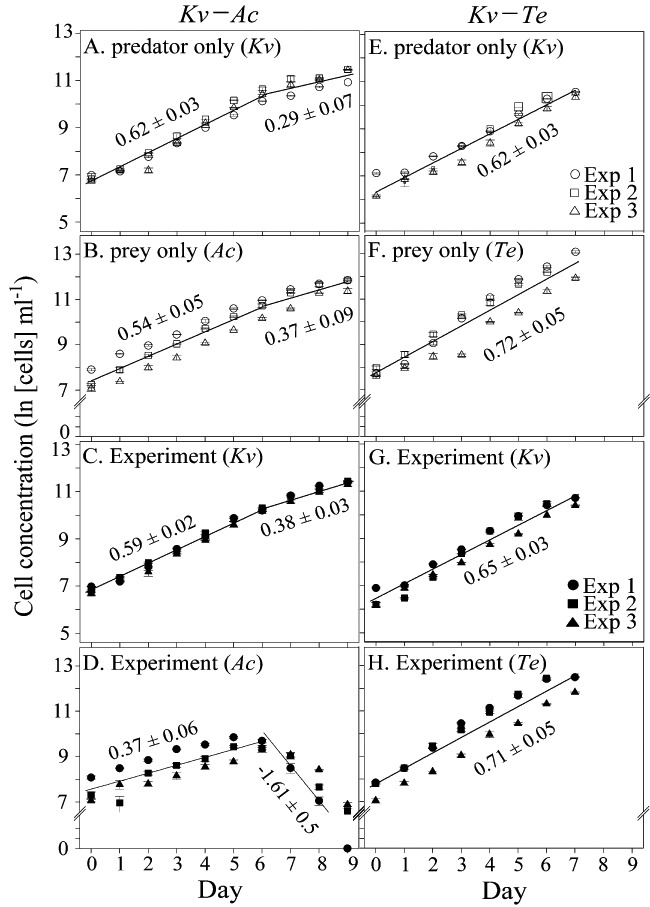
Concentrations of (A, E) *K. veneficum* in the predator controls; (B) *A. carterae* and (F) *Teleaulax* sp. in the prey controls; (C) *K. veneficum* and (D) *A. carterae* in treatments involving incubation of *K. veneficum* with *A. carterae* (*Kv–Ac*); and (G) *K. veneficum* and (H) *Teleaulax* sp. in treatments involving incubation of *K. veneficum* with *Teleaulax* sp. (*Kv–Te*). Different symbols represent different pseudo-replicate experiments, and open and filled symbols indicate the control and experimental bottles respectively. Error bars for cell concentrations (not clearly shown, as they are smaller than the symbols) indicate the differences of two measurements from the mean. The solid lines and numbers represent the best fits of data and growth rates respectively.

**Fig 2:**
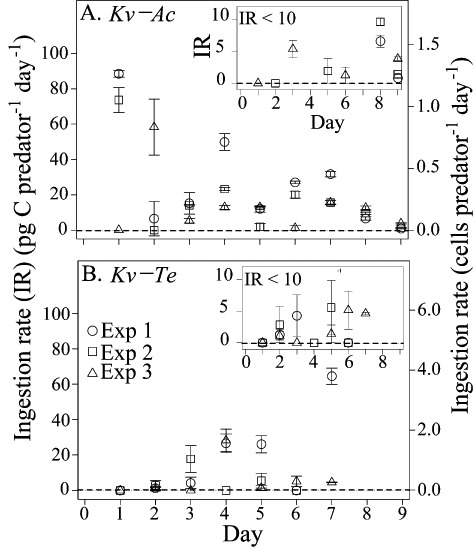
Ingestion rate (IR) (pg C predator^−1^ day^−1^) as a function of time (day) in experiment bottles containing (A) *K. veneficum* and *A. carterae* (*Kv–Ac*) or (B) *K. veneficum* and *Teleaulax* sp. (*Kv–Te*). Different symbols represent different pseudo-experiments. Error bars for ingestion rate indicate the differences of two measurements from the mean. The insets in (A) and (B) only show data with IR < 10 pg C predator ^−1^ day^−1^ to better visualize these data.

The mixotrophic nature of *K. veneficum* was also derived from sets of experiments involving two predator–prey pairs. In experiments involving *K. veneficum* and *A. carterae* as the predator–prey pair (*Kv*–*Ac*) (Fig. 1A–D), the ingestion rates of *A. carterae* by *K. veneficum* ranged from 0 to 90 pg C predator^–1^ day^–1^ (0 to 1.3 cells predator^–1^ day^–1^) (Fig. 2A; Table S1). In these experiments the abundance of *A. carterae* increased at rates approximately 35% lower than for this species in the prey-only controls; in three pseudo-replicate experiments the abundance of *A. carterae* in the experimental bottles began to decrease at day 6 after the experiment commenced (Fig. 1D). In experiments involving *K. veneficum* and *Teleaulax* sp. as the predator–prey pair (*Kv*–*Te*) (Fig. 1E–H), the ingestion rates of *Teleaulax* sp. by *K. veneficum* in the experimental bottles ranged from 0 to 70 pg C predator^–1^ day^–1^ (0–4 cells predator^–1^ day^–1^) (Fig. 2B; Table S2).

In an experimental bottle of a given pseudo-replicate experiment, the large day-to-day variations in ingestion rates were probably caused by a slight temporal decoupling of the ingestion of prey cells (i.e. grazing activity) and the cell division of the predator (i.e. the growth rate); these factors have opposite effects on the ingestion rate (which is proportional to the ratio of grazing activity to predator growth) (Jeong *et al*., 2010). In most cases, when feeding activity is high, the rate of cell division by the predator is low, and vice versa. For example, in experiment 1 involving the *Kv*–*Ac* pair (Exp 1 in Fig. 2A), the high ingestion rate during day 1 (90 pg C predator^–1^ day^–1^) was the result from the high number of prey cells ingested by the predator and the low rate of cell division of the predator. The sudden drop in the ingestion rate (from 90 to < 10 pg C predator^–1^ day^–1^) during day 2 on the other hand was the result of low number of prey cells ingested by the predator and the high rate of cell division of the predator. This phenomenon of day-to-day oscillations in the ingestion rates persisted throughout the experimental period, and was also observed in the other pseudo-replicate experiments. We also used different populations from the same culture of a dinoflagellate in all three pseudo-replicate experiments, because they were performed over a 3-month time period. Therefore, the physiology of one population used in the experimental bottle of a given pseudo-replicate experiment likely differed somewhat from that of the other populations used in the experimental bottles of the other two pseudo-replicate experiments. Such differences would account for the substantial variations observed in the ingestion rates among different pseudo-replicate experiments; for example, ∼0 versus 90 pg C predator^–1^ day^–1^ at day 1 in the *Kv*–*Ac* pair. However, the observed variations in ingestion rates in a given experimental bottle over time or among the three pseudo-replicate experimental bottles do not undermine the main conclusions drawn from the results.

### Cellular content of DMSP in the predator and prey

The cellular content of DMSP in phytoplankton varies considerably among algal taxa, and even within the same species there is considerable variability depending on environmental conditions including temperature, pH, CO_2_ concentration, nutrient status and solar radiation (Sunda *et al*., 2002; Stefels *et al*., 2007). Therefore, in experiments to assess the cellular content of DMSP in the three test species (one predator and two prey species), we kept these environmental factors constant. In particular, we bubbled air containing 420–450 ppmv CO_2_ through the culture solution during incubation to maintain the solution pH in the range 7.8–8.2. By doing so, the culture solution was maintained at a constant CO_2_ concentration. Without this control, the cellular DMSP content in the phytoplankton species tested would likely have changed during incubation (Sunda *et al*., 2002; Stefels *et al*., 2007). It should be noted that the use of f/4 medium for all culture solutions allowed the test phytoplankton species to grow without depletion of essential elements.

**Fig 3:**
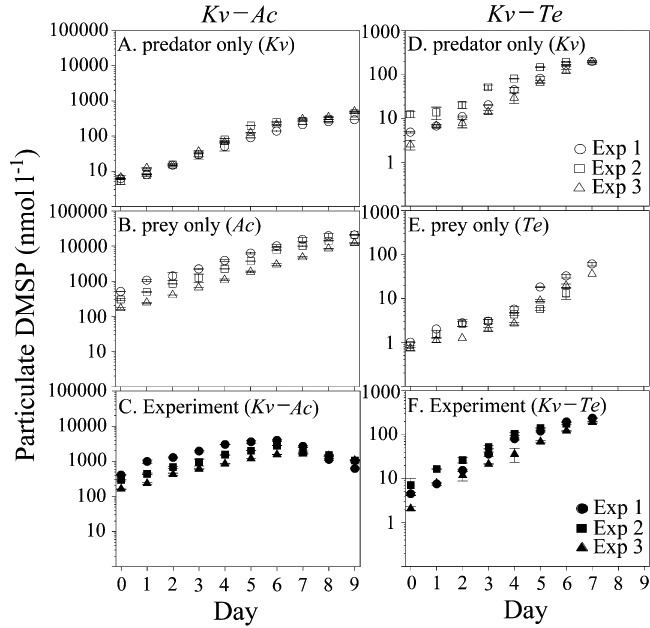
Particulate dimethylsulfoniopropionate (DMSP) concentrations as a function of time (day) (A, D) in the predator (*K. veneficum* alone) and (B, E) prey (*A. carterae* and *Teleaulax* sp. alone) controls, and in treatments involving incubation of (C) *K. veneficum* with *A. carterae* (*Kv–Ac*) and (F) *K. veneficum* with *Teleaulax* sp. (*Kv–Te*). Different symbols represent different pseudo-replicate experiments, and open and filled symbols indicate the control and experimental bottles respectively. Error bars for particulate DMSP (not clearly shown, as they are smaller than the symbols) indicate the standard deviations from the mean of replicate experiments.

**Fig 4:**
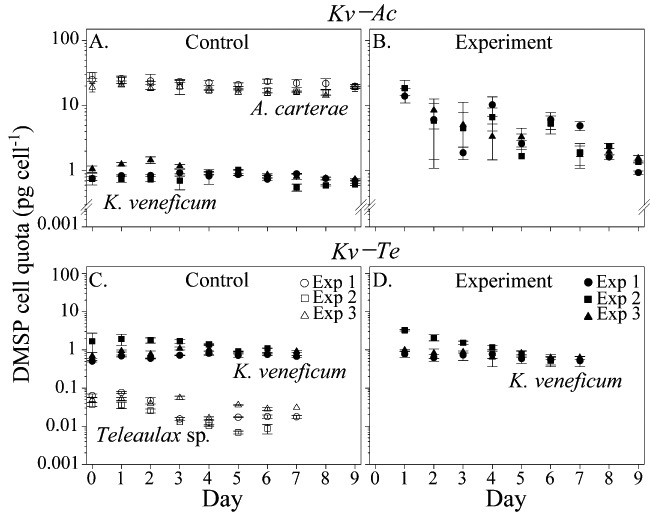
Evolution of the cellular contents of DMSP (pg cell^−1^) in *K. veneficum* (predator; filled symbols), and *A. carterae* and *Teleaulax* sp. (prey; open symbols) in the control bottles (A, C); and the cellular contents of DMSP (pg cell^−1^) in *K. veneficum* during mixotrophy in experimental bottles containing (B) *K. veneficum* and *A. carterae* (*Kv–Ac*) or (D) *K. veneficum* and *Teleaulax* sp. (*Kv–Te*). Different symbols represent three different pseudo-replicate experiments. Error bars for cellular contents of DMSP indicate the standard deviations from the mean of replicate measurements.

The cellular content of DMSP in *K. veneficum* (predator) and the prey species *A. carterae* and *Teleaulax* sp. was measured by dividing the particulate DMSP concentration in each control bottle (Fig. 3) by cell abundance. The content of DMSP in control cells of *K. veneficum* was 0.94 ± 0.19 pg DMSP cell^−1^ (1.30 ± 0.26 fg DMSP µm^−3^, i.e. femtograms of DMSP per µm^3^ cell volume) (Fig. 4A). For *A. carterae* the content was 19.7 ± 2.3 pg DMSP cell^−1^ (37.3 ± 4.5 fg DMSP µm^−3^) (Fig. 4A), which is approximately 20-fold greater (or 29-fold greater in terms of cell volume) than in *K. veneficum*. In contrast, for *Teleaulax* sp. the content was 0.03 ± 0.01 pg DMSP cell^−1^ (0.34 ± 0.11 fg DMSP µm^−3^) (Fig. 4C), which is two orders of magnitude lower (or fourfold lower in terms of cell volume) than in *K. veneficum*. During the experiment the content of DMSP in the control cells remained relatively constant for each species (within 10–30% of the mean values). We note that the cellular DMSP content (expressed per µm^3^ cell volume) in each of the three test species was calculated using measured cell volumes, as summarized in Table S3.

A key objective of our experiment was to assess how the mixotrophic nature of *K. veneficum* affects its cellular content of DMSP when it co-occurs with DMSP-rich (*A. carterae*) or DMSP-poor (*Teleaulax* sp.) prey. It was not convenient to directly measure the DMSP content of predator and prey cells in the experimental bottles because of difficulties associated with separating the cells of the different species. As an alternative, the cellular content of DMSP in *K. veneficum* in the experimental bottles ([DMSP]_PD_^EXP^) was determined on each sampling occasion by subtracting the DMSP contribution of prey cells (the number of prey in the experimental bottles, CELL_PR_^EXP^, multiplied by the cellular content of DMSP in prey in the prey-only control bottles, [DMSP]_PR_^CTL^) from the total particulate DMSP concentration in the experimental bottles ([DMSP^EXP^]), and dividing this value by the number of predator cells in the experimental bottles (CELL_PD_^EXP^), as described in the following equation:

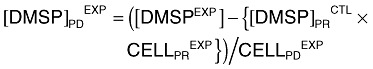
(1)

In this calculation we assumed that the cellular DMSP content in the prey species (*A. carterae* and *Teleaulax* sp.) in the experimental bottles was the same as in the prey controls ([DMSP]_PR_^CTL^). This assumption is critical in the case of *A. carterae*, because its cellular DMSP content was nearly 20 times higher than that of the predator. To assess the veracity of this assumption, we sorted 200–400 *A. carterae* cells from the experimental bottles containing the *Kv*–*Ac* pair, and we measured the particulate DMSP content of those sorted cells. The cellular DMSP content of *A. carterae* cells in the experimental bottles was indistinguishable (*t*-test; *P* = 0.497) from that in the control bottles (Table 1), indicating that the key assumption in our study was valid. In addition, the cell volumes of each of the three test species sampled from one control and the experimental bottles on days 0, 2, 4 and 6 (indicated as ‘Exp 1’ in Fig. 1) were measured to assess whether the cell volumes of the prey and predator changed as a result of grazing (Table S3). The mean cell volume of *K. veneficum* fed on *A. carterae* and *Teleaulax* sp. appeared to increase slightly, by 7 ± 3% and 11 ± 3% respectively. In contrast, we found no difference in the cell volume of either of the two prey species sampled from the control and experimental bottles (*t*-test; *P* = 0.668 for *Teleaulax* sp.; *P* = 0.670 for *A. carterae*).

**Table 1:** Cellular DMSP content (pg cell^−1^) for 200–400 *A. carterae* cells sorted from the *Ac*-control (CTL) and experimental (EXP) bottles containing *K. veneficum* and *A. carterae*.

	*Ac*-control bottles			*Kv–Ac* experimental bottles		
Batches	[DMSP]_Ac_^CTL^	SD	N	[DMSP]_Ac_^EXP^	SD^a^	N^b^
A	19.2	4.6	3	19.9	1.1	3
B	19.4	0.3	3	19.7	0.7	3
C	20.2	0.4	3	19.9	1.7	3
Mean	19.6	2.5	9	19.8	1.2	9

a Standard deviations from the mean of measurements.

b Number of measurements.

In experiments involving the *Kv*–*Ac* pair, the cellular DMSP content in *K. veneficum* in the experimental bottles ranged from 0.94 to 19.6 pg DMSP cell^−1^, which corresponded to 1–21 times the value found in the control bottles (Fig. 4B). In contrast, experiments involving the *Kv–Te* pair showed that a cellular DMSP content in *K. veneficum* was comparable to the content in the *K. veneficum*-control bottles (Fig. 4D). In particular, when the rates (1–65 pg C cell^−1^) at which *K. veneficum* ingested *Teleaulax* sp. were similar to the rates (1–88 pg C cell^−1^) at which *K. veneficum* ingested *A. carterae* during days 3–5 and 7, the enhancement of cellular DMSP content in *K. veneficum* due to mixotrophy in experiments involving the *K_V_–Te* pair was considerably smaller than that in *K. veneficum* in experiments involving the *K_V_–Ac* pair. This observation indicated that the DMSP content in the prey critically influenced the temporal variations in cellular DMSP content in the phototrophic dinoflagellate *K. veneficum* when growing mixotrophically.

### The fate of DMSP in prey during predation by mixotrophic *K. veneficum*

**Fig 5:**
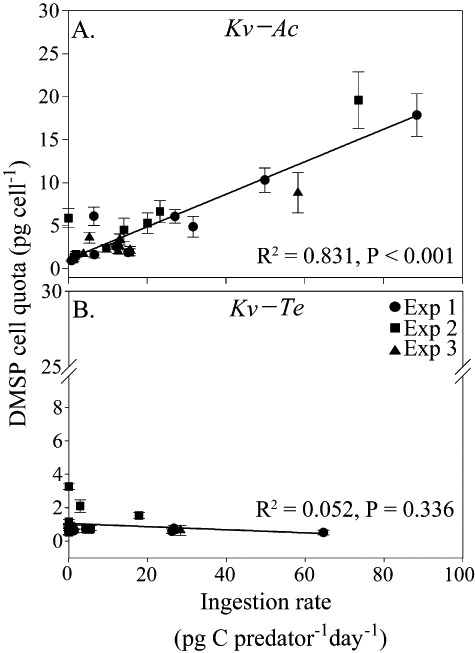
Cellular contents of DMSP (pg cell^−1^) in the predator *K. veneficum* during mixotrophy as a function of ingestion rate (pg C predator^−1^ day^−1^) with (A) *A. carterae* (*Kv–Ac*) and (B) *Teleaulax* sp. (*Kv–Te*) as prey. Different symbols represent different pseudo-replicate experiments. The solid lines in (A) and (B) are the best fits of data. Error bars for cellular contents of DMSP indicate the standard deviations from the mean of replicate measurements.

The day-to-day variations in the cellular content of DMSP in *K. veneficum* in the presence of the DMSP-rich *A. carterae* (see Fig. 4B) were linearly related to the corresponding ingestion rates (Fig. 5A). The high cellular content of DMSP in *K*. *veneficum* in experimental bottles during the initial days of the experiment declined rapidly, concurrently with a decrease in the ingestion rate. In particular, the DMSP cellular content in *K. veneficum* dropped close to the intrinsic value (the value observed in the *K. veneficum*-control bottles) when grazing rates decreased to nearly zero. Our results indicate that prey-derived DMSP appeared to be temporarily stored (for a few days) in the *K. veneficum* cells, after which point it was, likely, quickly transformed into other compounds. In contrast, the cellular content of DMSP in *K. veneficum* in the presence of the DMSP-poor *Teleaulax* sp. did not change with ingestion rate (Fig. 5B).

The amount of ingested DMSP retained in the predator cells was determined on each sampling occasion by dividing the cellular content of DMSP in *K. veneficum* in the experimental bottles at day *n* + 1 ([DMSP]_PD_^EXP^*_n_*_+1_) (obtained using Eq. 1) by the sum of the cellular content of DMSP in *K. veneficum* in the experimental bottles at day *n* ([DMSP]_PD_^EXP^*_n_*) and the total amount of DMSP ingested (ingestion rate, IR, multiplied by [DMSP]_PR_^CTL^) between day *n* and day *n* + 1, as described in the following equation:

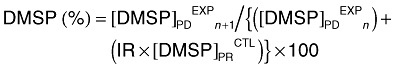
2

In our experiments using two predator–prey combinations, the predator retained 35–45% of the ingested DMSP in experiments involving the *Kv*–*Ac* pair, and 68–90% in experiments involving the *Kv–Te* pair (Table 2). The % DMSP retentions obtained for the experiments involving the *Kv–Te* pair included errors that were larger than those of experiments involving the *Kv*–*Ac* pair. In the experiments involving the *Kv*–*Te* pair, mixotrophy did not discernibly enhance the cellular DMSP content in *K. veneficum* due primarily to the low cellular DMSP content in *Teleaulax* sp. (i.e. three orders of magnitude lower than in *A. carterae*) and, to a lesser extent, to low grazing rates. As a result, errors arising from estimates of the rate at which *K. veneficum* grazed on *Teleaulax* sp. in the experimental bottles resulted in significant uncertainties in the % DMSP retention values (Table 2). The % DMSP retentions by the phototrophic *K. veneficum* during mixotrophy were consistent with the published values from experiments involving various microzooplankton (incapable of producing DMSP) grazing on DMSP-containing phytoplankton (e.g. Tang and Simó, 2003; Saló*et al*., 2009).

**Table 2:** Percentage DMSP retention by the predator *K. veneficum* in the experimental bottles containing *K. veneficum* and *A. carterae* (*Kv*–*Ac*) or *K. veneficum* and *Teleaulax* sp. (*Kv–Te*).

	% DMSP retention					
	*Kv–Ac*			*Kv–Te*		
Day	Exp. 1	Exp. 2	Exp. 3	Exp. 1	Exp. 2	Exp. 3
2	30 ± 7^a^	30 ± 8	–b	97 ± 15	64 ± 23	83 ± 51
3	17 ± 4	–	36 ± 11	95 ± 27	72 ± 31	–
4	57 ± 10	61 ± 22	39 ± 9	98 ± 29	–	91 ± 23
5	18 ± 4	25 ± 7	59 ± 14	72 ± 22	68 ± 16	96 ± 14
6	54 ± 10	75 ± 22	29 ± 7	–	–	75 ± 12
7	30 ± 8	20 ± 6	47 ± 10	76 ± 23	na^c^	99 ± 19
8	23 ± 8	53 ± 15	39 ± 8	na	na	na
9	50 ± 12	49 ± 13	58 ± 12	na	na	na
Mean ± SD	35 ± 16^d^	45 ± 20	44 ± 11	88 ± 13	68 ± 6	90 ± 11

a Errors associated with estimates of ingestion rate, measurements of intracellular DMSP content in *K. veneficum*, *A. carterae* and *Teleaulax* sp.

b ‘–’ indicates that grazing rates were not significantly different from zero.

c ‘na’ indicates no results available because the experiments involving the *Kv–Te* pair were terminated at day 7.

d Standard deviations from the mean of daily measurements.

Our results also indicate that significant amounts of the ingested DMSP were not retained. The fraction of ingested DMSP that was not retained was particularly significant for the *Kv*–*Ac* pair, but less so for the *Kv–Te* pair. The non-retained DMSP may be lost via several identified pathways: transformation to volatile DMS, release from grazing in a dissolved form and remained in a dissolved form, assimilation into other biochemical compounds (e.g. methionine) within the predator cell, and transformation of dissolved DMSP (released from grazing) into other compounds via bacterial demethylation (e.g. to methionine) or oxidation (e.g. to DMSO or SO_4_^2−^) processes. The first pathway (defined here as grazing-mediated DMS production) includes transformation of intracellular DMSP to DMS by intracellular DMSP-lyase enzymes via mixing of these two components during grazing and bacterial transformation into DMS of dissolved DMSP released during grazing. We note that in our experiments the DMSP present in the dissolved form (resulting from grazing and algal exudation) represented < 2% of the total ingested DMSP. The comparable concentrations of dissolved DMSP in the control (resulting from algal exudation only) and experimental (resulting from grazing and algal exudation) bottles indicate that most of the dissolved DMSP released during grazing was readily transformed into DMS or other compounds.

In our experiments to measure the retention of ingested DMSP, concurrent measurements of cellular DMSP content and DMS production were not possible because the continuous bubbling during incubation removed DMS from the culture solution. Therefore, to assess the involvement of grazing-mediated DMS production, we performed (in quadruplicate) a set of dilution experiments (100%, 75%, 50% and 25%), in which the rates of grazing-mediated DMS production were determined from the regression slope of the net DMS production rate as a function of the corresponding grazing rate for particulate DMSP multiplied by the mean concentration of particulate DMSP in the undiluted cultures (Table 3; Fig. S2; for more details of the experimental methods, see Wolfe *et al*., 2000;Saló*et al*., 2010). In this analysis, the intercept corresponded to the rate of net DMS production resulting from all other processes (i.e. algal exudation, cell lysis, bacterial cleavage of initial dissolved DMSP and bacterial DMS consumption). For example, in the second dilution experiment 32.2 nmol l^−1^ day^−1^ (11.3%) of the total particulate DMSP ingested (286.6 ± 16.1 nmol l^−1^ day^−1^) was found to be transformed into DMS as a result of grazing activity, of which 48.7% was retained as particulate DMSP and 2.5% remained as dissolved DMSP (Table 3). The remaining 37.5% could have entered three pathways: assimilation into other compounds within the predator cell, transformation of dissolved DMSP (released during grazing) into other compounds via bacterial demethylation, and oxidation processes. However, we were unable to quantify the partitioning of the remainder among these pathways because not all the compounds resulting from these processes were measured. In this experiment, the grazing-mediated DMS production (32.2 ± 1.0 nmol l^−1^ day^−1^) accounted for 66% of the gross production (48.8 ± 0.6 nmol l^−1^ day^−1^), which was determined from the inhibitor experiment (see detailed description of the method in *Experimental procedures*), with the other processes being responsible for the remaining DMS (Table 4). In three other dilution experiments the DMS production resulting from grazing activity was also a major contributor to gross DMS production. The results of the experiments described above (the dilution and inhibitor experiments) indicated that the DMS production observed in the experimental bottles probably resulted from grazing activity.

**Table 3:** The fates of ingested DMSP in *A. carterae* during predation by mixotrophic *K. veneficum*, determined using dilution experiments.

DMSP/DMS/other compounds (nmol l^−1^ day^−1^)	Dilution Exp. 1	Dilution Exp. 2	Dilution Exp. 3	Dilution Exp. 4
Grazed DMSP	119.6 ± 9.1^a^	286.6 ± 16.1	732.6 ± 53.5	528.0 ± 28.0
Retained DMSP	39.5 ± 9.9	139.6 ± 32.7	322.4 ± 36.6	281.4 ± 82.4
	(33.0%)^b^	(48.7%)	(44.0%)	(53.3%)
Grazing-mediated DMS production	7.3 ± 1.5	32.2 ± 1.0	35.8 ± 3.4	29.0 ± 0.6
	(6.1%)^c^	(11.3%)	(4.9%)	(5.5%)
Net dissolved DMSP production	2.3 ± 0.5	7.2 ± 2.0	10.5 ± 2.2	6.3 ± 1.1
	(< 1.9%)^d^	(< 2.5%)	(< 1.4%)	(< 1.2%)
Other compounds^e^	∼70.5^f^	∼107.6	∼363.9	∼211.3
	(60.9%)	(37.5%)	(49.7%)	(40.0%)

a One standard deviation from replicate measurements.

b Percentage of retained DMSP relative to the total DMSP grazed.

c Percentage of grazing-mediated DMS production relative to the total DMSP grazed.

d Percentage of net dissolved DMSP production relative to the total DMSP grazed.

e Includes assimilation into other biochemical compounds within the predator cell, and transformation into other compounds via bacterial demethylation or oxidation processes.

f Percentage of other compounds (e.g. methionine, DMSO and SO_4_^2−^) relative to the total DMSP grazed.

**Table 4:** DMS production rates (nmol l^−1^ day^−1^) determined from the dilution and inhibition experiments.

DMS				
(nmol l^−1^ day^−1^)	Dilution Exp. 1	Dilution Exp. 2	Dilution Exp. 3	Dilution Exp. 4
Gross production	10.5 ± 0.1^a^	48.8 ± 0.6	40.9 ± 1.3	35.7 ± 1.1
Grazing-mediated production	7.3 ± 1.5	32.2 ± 1.0	35.8 ± 4.8	29.0 ± 0.6
Production other than grazing^b^	3.6 ± 0.9	19.2 ± 2.6	0.3 ± 0.7	7.9 ± 1.3
Bacterial consumption	5.6 ± 0.1	10.2 ± 1.2	7.7 ± 2.0	3.9 ± 0.6

a One standard deviation from replicate measurements.

b The sum of the net DMS production resulting from all other processes (slopes in Fig. S2) and bacterial DMS consumption.

Intracellular DMSP in marine dinoflagellates generally accounts for 10–90% of the total cell sulfur (Simó*et al*., 2002; 2009) and up to 10% of the total cell carbon (Stefels *et al*., 2007). The mixotrophic dinoflagellate *K. veneficum* produce DMSP by photosynthesis (see Fig. 3A and D), but the present study found that it also acquires DMSP by grazing on DMSP-containing algal species (Fig. 4B). In contrast with the former mechanism, the latter is likely to conserve some of the metabolic energy used to produce this compound. *Karlodinium veneficum* may satisfy some of its carbon demand via phagotrophy, and the simultaneous DMSP acquisition could be a result of this phagotrophy. Therefore, acquiring the essential elements C and S via phagotrophy may provide dinoflagellates with a competitive advantage over strictly photosynthetic and heterotrophic algae (Bockstahler and Coats, 1993).

An additional finding worthy of discussion is that *K. veneficum* preferred grazing on DMSP-rich prey over DMSP-poor prey, which contrasts with the results of previous studies (e.g. Wolfe *et al*., 1997; Storm *et al*., 2003). In those studies, grazing by heterotrophic dinoflagellates was deterred by the presence of acrylate formed from the cleavage of DMSP. However, results from a recent study showed that in some plankton species, DMSP acted as an attractant to prey rather than as a deterrent (Seymour *et al*., 2010), which is in line with our results. Because several other factors (predator and prey cell size, cell surface properties and release of dissolved chemical cues) are also involved in the selective feeding among prey species (Montagnes *et al*., 2008; Roberts *et al*., 2011), it is not possible to draw firm conclusions about selective feeding. Addressing this issue is the beyond the scope of the present study.

Our results indicate a new pathway by which significant amounts of DMS could be produced when phototrophic dinoflagellates feed on DMSP-rich prey cells. The results obtained in this study are similar to those obtained from experiments involving microzooplankton grazing on DMSP-containing prey (e.g. Tang and Simó, 2003; Saló*et al*., 2009), although the exact grazing mechanisms may differ between the phototrophic and heterotrophic dinoflagellates. Our results have an important geochemical implication in the marine cycles of DMSP and DMS. Recent studies have shown that red tide blooms in coastal waters (usually caused by dinoflagellates) produce significant quantities of DMS, which is a precursor of naturally occurring methanesulfonic acid (MSA, which has no anthropogenic source) that may lead to the formation of aerosol (Gaston *et al*., 2010). As mixotrophic and heterotrophic dinoflagellates are major grazers in coastal bloom waters and in upwelling areas (Strom and Strom, 1996; Jeong *et al*., 2005), the mixotrophic nature of dinoflagellates could enhance DMS production in these waters.

## Experimental section

### Culture of experimental organisms

Dense cultures (10^4^–10^5^ cells ml^−1^) of *A. carterae*, *Teleaulax* sp. and *K. veneficum* (isolated from the western coastal waters of Korea) were grown for approximately 2 weeks. Appropriate volumes of the dense cultures (100–200 ml) were added to 10 l polycarbonate bottles containing 4 l of f/2 medium (Guillard and Ryther, 1962) without silicate and 4 l of seawater (filtered through a 0.2 µm filter). The f/2 medium and filtered seawater were autoclaved prior to addition to the 10 l bottles. We installed one acryl pillar (30 mm diameter and 310 mm high) in each culture bottle to gently aerate the seawater with air containing 420–450 ppmv CO_2_ (100 ml min^−1^). As fine bubbles from the air stone raised seawater from the lower part of the bottle to the surface, outside seawater was introduced into the pillar through holes immediately below the air stone. Consequently, incoming seawater was continuously transferred to the surface by the rising bubbles. This mixing scheme generated a convective flow of seawater within the bottle that enhanced the homogeneity of the seawater in terms of phytoplankton cell density and solute concentration. The standard deviations from the average of the estimations of phytoplankton cell abundances and from the DMSP concentrations (in triplicate samples) were less than 10% and 5% respectively. The initial cell concentrations were approximately 500–1000 cells ml^−1^ for the predator (*K. veneficum*) and 1000–2000 cells ml^−1^ for each of the prey species (*A. carterae* and *Teleaulax* sp.). We established two experimental treatments comprising mixtures of *A. carterae* with *K. veneficum* and *Teleaulax* sp. with *K. veneficum*, and controls comprising *K. veneficum*, *A. carterae* and *Teleaulax* sp. alone. The control and experimental bottles were placed at 20°C under a 12:12 h light–dark cycle, using cool white fluorescent lights (75 µmol photons m^−2^ s^−1^).

### Cell enumeration

To determine the cell densities (cells ml^−1^) of predator and prey, a 15 ml aliquot was removed from each bottle at each sampling time, and was immediately fixed with 5% (v/v) Lugol's solution. The different species were enumerated in 1 ml of samples in Sedgwick-Rafter counting chambers, based on triplicate counts of more than 300 cells.

### Ingestion rates

On each sampling occasion, the ingestion rates (IR, pg C predator^−1^ day^−1^ or prey cells predator^−1^ day^−1^) of *A. carterae* and *Teleaulax* sp. by *K. veneficum* in the experimental bottles were measured according to the concentrations of predator and prey in the control and experimental bottles sampled between days *n* and *n* + 1 of incubation, as described by the following equation (Frost, 1972; Jeong *et al*., 2005):

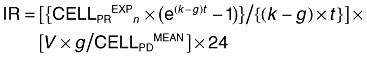
3

where the first term is the mean prey concentration (ng C ml^−1^), the second term is the clearance rate (ml predator^−1^ h^−1^), and the numeric value indicates the number of hours. In the first term in Eq. 3, CELL_PR_^EXP^*_n_* is the prey concentration in the experimental bottles at day *n*; and *k* ([ln CELL_PR_^CTL^*_n_*_+1_/CELL_PR_^CTL^*_n_*]/*t*) is the prey growth constant (h^−1^), where CELL_PR_^CTL^*_n_*_+1_ is the prey concentration in the control bottles at day *n* + 1 and *t* is hours of incubation between days *n* and *n* + 1. In the second term, *V* is the bottle volume (equal to the volume of the incubation solution sampled, ml), *g* (*k* − ln[CELL_PR_^EXP^*_n_*_+1_/CELL_PR_^EXP^*_n_*]/*t*) is the grazing constant (h^−1^), and CELL_PD_^MEAN^ (CELL_PD_^EXP^*_n_* × [e*^µ^*^×^*^t^* − 1]/[*µ* × *t*]) is the mean predator concentration in the experimental bottles, where CELL_PD_^EXP^*_n_* is the concentrations of predator in the experimental bottles at day *n* and *µ* is the growth rate of the predator in the experiment bottles.

### Transmission electron microscopy

Transmission electron microscopy (TEM) analysis confirmed predation by *K. veneficum* on *A. carterae.* Predator and prey cells were incubated at 20°C in a 270 ml polycarbonate bottle. The initial cell concentrations in the experimental bottle were approximately 30000 cells ml^−1^ for *A. carterae* and 5000 cells ml^−1^ for *K. veneficum*. The experimental bottle was placed on a plate which was vertically rotated at 0.9 r.p.m. and maintained at 20°C under a 14:10 h light–dark cycle of cool white fluorescent light at 20 µmol photons m^−2^ s^−1^. A 50 ml aliquot from the experimental bottle (containing *K. veneficum* and *A. carterae*) was sampled at day 3 after incubation had commenced. Cells were centrifuged, and the pellet was embedded in 1% agar (w/v). After several rinses with culture medium, the cells were post-fixed in 1% (w/v) osmium tetroxide in deionized water and then dehydrated using a graded ethanol series [50%, 60%, 70%, 80%, 90% and 100% (all v/v) ethanol, followed by two washes with 100% ethanol]. The cells were then embedded in Spurr's low viscosity resin (Spurr, 1969), sectioned using an RMC MT-XL ultramicrotome (Boeckeler Instruments, USA), and post-stained with 3% (w/v) aqueous uranyl acetate followed by lead citrate. Stained sections were viewed with a JEOL-1010 electron microscope (Jeol, Japan).

### DMSP and DMS analyses

Samples for DMSP analysis were collected and preserved as described in Kiene and Slezak (2006). To determine the particulate DMSP concentration, we measured the total DMSP (dissolved and particulate forms) and dissolved DMSP concentrations (Fig. S3) in the experimental bottles. The particulate DMSP concentration was calculated as the difference between these values. Total DMSP analysis was accomplished by adding small amounts of 50% H_2_SO_4_ (5 µl per ml sample) to the samples (∼10 ml) for preservation until analysis. Dissolved DMSP analysis was accomplished by gravitationally filtering the seawater samples through a GF/F filter (47 mm in diameter) using the small volume drip filtration procedure. The samples were then preserved by addition of 50% H_2_SO_4_ (5 µl addition per ml sample) until analysis. The DMSP samples were then hydrolysed to DMS using 10 N NaOH (addition of 0.25 ml per ml sample) and allowed to react overnight in the dark. Subsequently evolving DMS was measured by gas chromatography using flame photometric detector (GC-FPD).

DMS analysis was conducted by gravitationally filtering 30 ml of samples through a GF/F filter (47 mm in diameter). Filtrates were stored in an amber glass vial with no headspace, and the vial was quickly sealed with a gas-tight cap, the inside of which was coated with Teflon. Within an hour of sampling, 2–20 ml of samples were delivered to the sparging chamber to measure the DMS concentration.

For both DMSP and DMS analysis, the GC-FPD was calibrated against standard DMS solutions of known concentration prepared by alkaline hydrolysis of DMSP-Cl (Tokyo Kasei) in an amber vial (30 ml) with a gas-tight Teflon cap. The response of the GC-FPD was also independently calibrated against gas standards with certified mole fractions of a DMS gas standard (Scott Specialty, 3410 ppbv DMS). Detailed descriptions of the DMS and DMSP analysis have been reported elsewhere (Park and Lee, 2008).

### Dilution and inhibitor experiments

A series of dilutions (100%, 75%, 50% and 25%) was prepared in 1.2 l polycarbonate bottles by adding particle-free seawater enriched with f/4 medium to the experimental bottle solution containing the *Kv*–*Ac* pair (for more details, see Wolfe *et al*., 2000; Saló*et al*., 2010). The experiment was performed in duplicate and no headspace was retained in the bottles. In a parallel experiment we prepared two sets of undiluted (100%) solution in 1.2 l polycarbonate bottles (also no headspace): to one set we added 50 µl of chloroform (final chloroform concentration 500 µM) as an inhibitor of bacterial DMS consumption, and the other set had no added chloroform (for a more detailed description of the method, see Kiene and Bates, 1990). All the dilution and inhibitor bottles were incubated for 24 h.

Aliquots (∼150 ml) for cell enumeration and DMS and DMSP analysis were removed from the dilution bottles immediately as well as after 24 h. Within each dilution sample, the difference between DMS concentrations measured at incubation times *t* = 0 and 24 equalled to the net DMS production. At each dilution level, the rate of DMSP grazing was obtained by multiplying the DMSP mortality rate by the appropriate dilution factor and the mean DMSP concentration. The mean DMSP concentration in each replication was calculated using the equation presented in Frost (1972). We then plotted values of the net DMS production rate against the corresponding DMSP grazing rate. The regression slope describing the relationship between these two parameters indicated the daily DMS production per unit DMSP grazed. The rate of DMS production arising solely from grazing activity would have been obtained if the regression slope had been multiplied by the mean DMSP concentration in the non-diluted bottles. Aliquots (∼15 ml) for DMS analysis were removed from each inhibitor bottle at incubation times *t* = 0, 8 and 24. The DMS concentrations measured in the bottles to which chloroform was added were plotted as a function of incubation time; the resulting slops represented the rate of gross DMS production. The slopes determined from sampling of the bottles without added chloroform equated to the net DMS production rate. The difference was equal to the rate of bacterial DMS consumption.

### Seawater pH

Measurements of seawater sample pH were made at 25°C using a double wavelength spectrophotometric procedure and thymol blue indicator, following the procedure described by Zhang and Byrne, (1996). The measurements were made to a precision of ±0.002 in pH (Kim and Lee, 2009).

## Supporting information

Additional Supporting Information may be found in the online version of this article: **Fig. S1.** Feeding process of *K. veneficum*(*Kv*) on *A. carterae* (*Ac*). (A) *K.veneficum* encountering *A. carterae* and (B) *K.veneficum* ingesting the prey cytoplasm (marked as white arrows)through the peduncle and transferring a prey cell to a food vacuoleinside the protoplasm of *K. veneficum* through the peduncle.Transmission electron microscopy images of (C) *K. veneficum*,(D) *A. carterae*, (E) *K. veneficum* with an ingested*A. carterae* cell, and (F) enlargement of the ingested *A.carterae* cell. Scale bars shown in (A)?(B), (C)?(E) and (F)represent 5 µm, 1 µm and 200 nm respectively. 'pdc' and'pc' in (C)?(F) mean predator and prey chloroplastrespectively. **Fig. S2.** Relationships between the amount ofDMS produced and the amount of DMSP grazed at each dilution levelin four dilution experiments in which *K. veneficum* fed on*A. carterae.* The vertical error bars for net DMS production(*y*-axis) and the horizontal error bars for the grazing rateof DMSP (*x*-axis) indicate the standard deviations from themean of replicate measurements. The slope represents the daily DMSproduction (nmol l^−1^ day^−1^) pergrazed DMSP (nmol l^−1^ day^−1^). **Fig. S3.** Dissolved dimethylsulfoniopropionate(DMSP) concentrations as a function of time (day) (A, D) in thepredator (*K. veneficum* alone) and (B, E) prey (*A.carterae* and *Teleaulax* sp. alone) controls, and intreatments involving incubation of (C) *K. veneficum* with*A. carterae* (*Kv?Ac*) and (F) *K. veneficum* with*Teleaulax* sp. (*Kv?Te*). Different symbols representdifferent pseudo-replicate experiments, and open and filled symbolsindicate the control and experimental bottles respectively. **Table S1.** Specific growth rates(day^−1^) of *K. veneficum* and *A.carterae* in controls, and in experimental treatments involvingincubation of *K. veneficum* with *A. carterae*(*Kv?Ac*), and ingestion rates (IR, pg Cpredator^−1^ day^−1^) of *K.veneficum* in experimental treatments. Values in parenthesis indicate errors. **Table S2.** Specific growth rates(day^−1^) of *K. veneficum* and*Teleaulax* sp. in controls, and in experimental treatmentsinvolving incubation of *K. veneficum* with *Teleaulax*sp. (*Kv?Te*), and ingestion rates (IR, ng Cpredator^−1^ day^−1^) of *K.veneficum* in experimental treatments. Values in parenthesis indicate errors. **Table S3.** Cell volumes (µm^3^)of *K. veneficum*, *Teleaulax* sp. and *A. carterae* in the control (CTL) and experimental (EXP) bottles in one of the replicate experiments shown in Fig. 1 (labelled as EXP 1).

## References

[b1] Adolf JE, Stocker DK, Harding LW (2006). The balance of autotrophy and heterotrophy during mixotrophic growth of *Karlodinium micrum* (Dinophyceae). J Plankton Res.

[b2] Adolf JE, Bachvaroff T, Place AR (2008). Can cryptophytes abundance trigger toxic *Karlodinium veneficum* blooms in eutrophic estuaries?. Harmful Algae.

[b3] Bockstahler KR, Coats DW (1993). Spatial and temporal aspects of mixotrophy in Chesapeake Bay dinoflagellates. J Eukaryot Microbiol.

[b4] Burkill PH, Archer SD, Robinson C, Nightingale PD, Groom SB, Tarran GA, Zubkov MV (2002). Dimethyl sulphide biogeochemistry within a coccolitophore bloom (DISCO): an overview. Deep Sea Res II.

[b5] Charlson RJ, Lovelock JE, Andreae MO, Warren SG (1987). Oceanic phytoplankton, atmospheric sulphur, cloud albedo and climate. Nature.

[b6] Dacey JWH, Blough NV (1987). Hydroxide decomposition of dimethylsulfoniopropionate to form dimethylsulfide. Geophys Res Lett.

[b7] Frost BW (1972). Effects of size and concentration of food particles on the feeding behaviour of the marine planktonic copepod *Calanus pacificus*. Limnol Oceanogr.

[b8] Gaston CJ, Pratt KA, Qin X, Prather KA (2010). Real-time detection and mixing state of methanesulfonate in single particles at an inland urban location during a phytoplankton bloom. Environ Sci Technol.

[b9] Guillard RR, Ryther JH (1962). Studies of marine planktonic diatoms. I. *Cyclotella nana* Hustedt and *Detonula confervacea* (Cleve) Gran. Can J Microbiol.

[b10] Jacobson DM, Anderson DM (1996). Widespread phagocytosis of ciliates and other protists by marine mixotrophic and heterotrophic thecate dinoflagellates. J Phycol.

[b11] Jeong HJ, Yoo YD, Kim JS, Kim TH, Kim JH, Kang NS, Yih WH (2004). Mixotrophy in the phototrophic harmful alga *Cochlodinium polykrikoides* (Dinophycean): prey species, the effects of prey concentration and grazing impact. J Eukaryot Microbiol.

[b13] Jeong HJ, Yoo YD, Park JY, Song JY, Kim ST, Lee SH (2005). Feeding by the phototrophic red-tide dinoflagellates: five species newly revealed and six species previously known to be mixotrophic. Aquat Microb Ecol.

[b14] Jeong HJ, Yoo YD, Kim JS, Seong KA, Kang NS, Kim TH (2010). Growth, feeding and ecological roles of the mixotrophic and heterotrophic dinoflagellates in marine planktonic food webs. Ocean Sci J.

[b16] Keller MD, Bellows WK, Guillard RRL, Saltzman ES, Cooper EM (1989). Dimethylsulfide production in marine phytoplankton. Biogenic Sulfur in the Environment.

[b15] Kettle AJ, Andreae MO (2000). Flux of dimethylsulfide from the oceans: a comparison of updated data set and flux models. J Geophys Res.

[b17] Kiene RP, Bates TS (1990). Biological removal of dimethylsulfide from sea water. Nature.

[b18] Kiene RP, Slezak D (2006). Low dissolved DMSP concentrations in seawater revealed by small-volume gravity filtration and dialysis sampling. Limnol Oceanogr Methods.

[b19] Kim H-C, Lee K (2009). Significant contribution of dissolved organic matter to seawater alkalinity. Geophys Res Lett.

[b20] Kirst GO, Thiel C, Wolff H, Nothnagel J, Wanzek M, Ulmke R (1991). Dimethylsulfoniopropionate (DMSP) in ice – algae and its possible biological role. Mar Chem.

[b21] Li A, Stoecker DK, Coats DW, Adam EJ (1996). Ingestion of fluorescently-labeled and phycoerythrin-containing prey by photosynthetic dinoflagellates. Aquat Microb Ecol.

[b22] Li A, Stoecker DK, Adolf JE (1999). Feeding, pigmentation, photosynthesis and growth of the mixotrophic dinoflagellate *Gyrodinium galatheanum*. Aquat Microb Ecol.

[b23] Li A, Stoecker DK, Coats DW (2000). Mixotrophy in *Gyrodinium galatheanum* (Dinophyceae): grazing responses to light intensity and inorganic nutrients. J Phycol.

[b24] Lovelock JE, Maggs RJ, Rasmussen RA (1972). Atmospheric sulphur and the natural sulphur cycle. Nature.

[b25] Malin G, Wilson WH, Bratbak G, Liss PS, Mann NH (1998). Elevated production of dimethylsulfide resulting from viral infection of cultures of *Phaeocystis pouchetii*. Limnol Oceanogr.

[b26] Matrai P, Keller MD (1993). DMS in a large scale coccolithophore bloom in the Gulf of Maine. Cont Shelf Res.

[b27] Montagnes DJS, Barbosa AB, Boenigk J, Davidson K, Jürgens K, Macek M (2008). Selective feeding behavior of key free-living protists: avenues for continued study. Aquat Microb Ecol.

[b28] Park K-T, Lee K (2008). High-frequency, accurate measurement of dimethylsulfide in surface marine environments using a microporous membrane contactor. Limnol Oceanogr Methods.

[b29] Roberts E, Wooton E, Davidson K, Jeong HJ, Lowe CD, Montagnes DJS (2011). Feeding in the dinoflagellate *Oxyrrhis marina*: linking behaviour with mechanisms. J Plankton Res.

[b30] Saló V, Simó R, Vila-Costa M, Calbet A (2009). Sulfur assimilation by *Oxyrrhis marina* feeding on a ^35^S-DMSP-labelled prey. Environ Microbiol.

[b31] Saló V, Simó R, Calbet A (2010). Revisiting the dilution technique to quantify the role of microzooplankton in DMS(P) cycling: laboratory and field tests. Environ Microbiol.

[b32] Seymour JR, Simó R, Ahmed T, Stocker R (2010). Chemoattraction to dimethylsulfoniopropionate throughout the marine microbial food web. Science.

[b33] Sheng J, Malkiel E, Katz J, Adolf JE, Place AR (2010). A dinoflagellate exploits toxins to immobilize prey prior to ingestion. Proc Natl Acad Sci USA.

[b35] Simó R, Archer S, Pedrós-Alió C, Gilpin L, Stelfox-Widdicombe CE (2002). Coupled dynamics of dimethylsulfoniopropionate and dimethylsulfide cycling and the microbial food web in surface waters of the North Atlantic. Limnol Oceanogr.

[b36] Simó R, Vila-Costa M, Alonso-Sáez L, Cardelús C, Guadayol Ò, Vázquez-Domínguez E, Gasol JM (2009). Annual DMSP contribution to S and C fluxes through phytoplankton and bacterioplankton in a NW Mediterranean coastal site. Aquat Microb Ecol.

[b37] Spurr AR (1969). A low viscosity epoxy resin embedding medium for electron microscopy. J Ultrastruct Res.

[b39] Stefels J, Steinke M, Turner S, Malin G, Belviso S (2007). Environmental constraints on the production and removal of the climatically active gas dimethylsulphide (DMS) and implications for ecosystem modeling. Biogeochemistry.

[b40] Steinke M, Malin G, Archer SD, Burkill PH, Liss PS (2002). DMS production in a coccolithophorid bloom: evidence for the importance of dinoflagellate DMSP lyases. Aquat Microb Ecol.

[b38] Stoecker DK (1999). Mixotrophy among dinoflagellates. J Eukaryot Microbiol.

[b42] Storm S, Wolfe G, Slajer A, Lambert S, Clough J (2003). Chemical defense in the micorplankton II: inhibition of protist feeding by β-dimethylsulfoniopropionate (DMSP). Limnol Oceanogr.

[b41] Strom SL, Strom MW (1996). Microplankton growth, grazing, and community structure in the northern Gulf of Mexico. Mar Ecol Prog Ser.

[b43] Sunda W, Kieber DJ, Kiene RP, Huntsman S (2002). An antioxidant function for DMSP and DMS in marine algae. Nature.

[b44] Tang KW, Simó R (2003). Trophic uptake and transfer of DMSP in simple planktonic food chain. Aquat Microb Ecol.

[b45] Todd JD, Rogers R, Li YG, Wexler M, Bond PL, Sun L (2007). Structural and regulatory genes required to make the gas dimethyl sulfide in bacteria. Science.

[b46] Wolfe GV, Steinke M (1996). Grazing-activated production of dimethylsulfide (DMS) by two clones of *Emiliania huxleyi*. Limnol Oceanogr.

[b47] Wolfe GV, Steinke M, Kirst GO (1997). Grazing-activated chemical defense in a unicellular marine alga. Nature.

[b48] Wolfe GV, Levasseur M, Cantin G, Michaud S (2000). DMSP and DMS dynamics and microzooplankton grazing in the Labrador Sea: application of the dilution technique. Deep Sea Res.

[b49] Zhang H, Byrne RH (1996). Spectrophotometric pH measurements of surface seawater at *in situ* conditions: absorbance and protonation behavior of thymol blue. Mar Chem.

[b50] Zubkov MV, Fuchs BM, Archer SD, Kiene RP, Amann R, Burkill PH (2001). Linking the composition of bacterioplankton to rapid turnover of dissolved dimethylsulfoniopropionate in an algal bloom in the North Sea. Environ Microbiol.

