# Supplementary material for: Prey-dependent retention of dimethylsulfoniopropionate (DMSP) by mixotrophic dinoflagellates
Source: Environ Microbiol. 2012 Mar;14(3):605–16. doi: 10.1111/j.1462-2920.2011.02600.x (PMC3490370; doi:10.1111/j.1462-2920.2011.02600.x)
Supplement: Supplementary file 1 — Additional Supporting Information may be found in the online version of this article: Fig. S1. Feeding process of K. veneficum(Kv) on A. carterae (Ac). (A) K.veneficum encountering A. carterae and (B) K.veneficum ingesting the prey cytoplasm (marked as white arrows)through the peduncle and transferring a prey cell to a food vacuoleinside the protoplasm of K. veneficum through the peduncle.Transmission electron microscopy images of (C) K. veneficum,(D) A. carterae, (E) K. veneficum with an ingestedA. carterae cell, and (F) enlargement of the ingested A.carterae cell. Scale bars shown in (A)?(B), (C)?(E) and (F)represent 5 µm, 1 µm and 200 nm respectively. 'pdc' and'pc' in (C)?(F) mean predator and prey chloroplastrespectively. Fig. S2. Relationships between the amount ofDMS produced and the amount of DMSP grazed at each dilution levelin four dilution experiments in which K. veneficum fed onA. carterae. The vertical error bars for net DMS production(y-axis) and the horizontal error bars for the grazing rateof DMSP (x-axis) indicate the standard deviations from themean of replicate measurements. The slope represents the daily DMSproduction (nmol l−1 day−1) pergrazed DMSP (nmol l−1 day−1). Fig. S3. Dissolved dimethylsulfoniopropionate(DMSP) concentrations as a function of time (day) (A, D) in thepredator (K. veneficum alone) and (B, E) prey (A.carterae and Teleaulax sp. alone) controls, and intreatments involving incubation of (C) K. veneficum withA. carterae (Kv?Ac) and (F) K. veneficum withTeleaulax sp. (Kv?Te). Different symbols representdifferent pseudo-replicate experiments, and open and filled symbolsindicate the control and experimental bottles respectively. Table S1. Specific growth rates(day−1) of K. veneficum and A.carterae in controls, and in experimental treatments involvingincubation of K. veneficum with A. carterae(Kv?Ac), and ingestion rates (IR, pg Cpredator−1 day−1) of K.veneficum in experimental treatments. Values in parenthesis indicate errors. Table [file emi0014-0605-SD1.doc]

**Supplementary materials**

**Figure S1.** Feeding process of *K. veneficum* (*Kv*) on *A. carterae* (*Ac*). (A) *K. veneficum* encountering *A. carterae* and (B) *K. veneficum* ingesting the prey cytoplasm (marked as white arrows) through the peduncle and transferring a prey cell to a food vacuole inside the protoplasm of *K .veneficum* through the peduncle. Transmission electron microscopy images of (C) *K. veneficum*, (D) *A. carterae*, (E) *K. veneficum* with an ingested *A. carterae* cell, and (F) enlargement of the ingested *A. carterae* cell. Scale bars shown in (A, B), (C, D, E) and (F) represent 5 μm, 1 μm and 200 nm, respectively. “pdc” and “pc” in (C, D, E and F) mean predator and prey chloroplast, respectively.


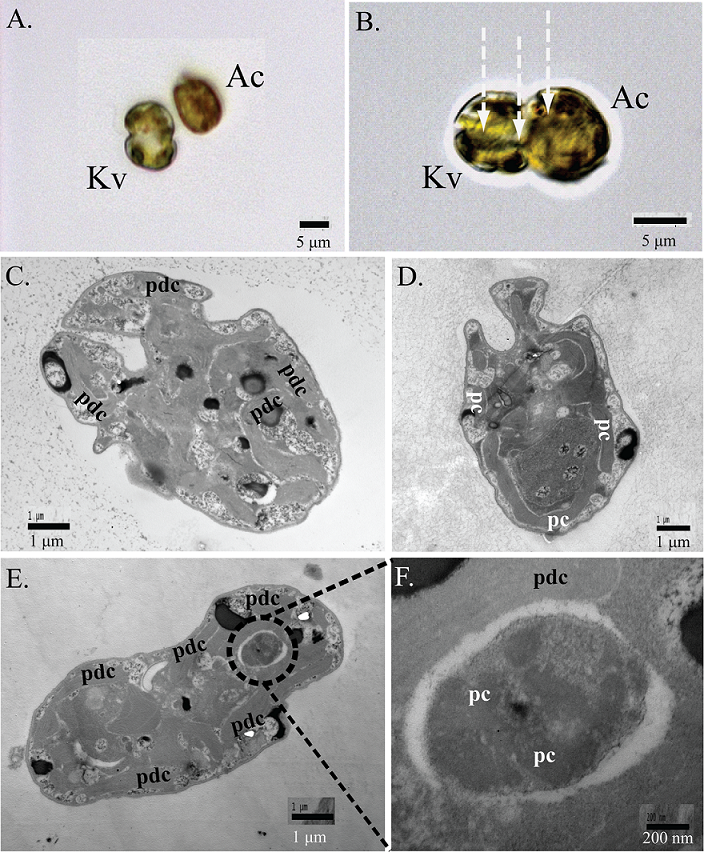


**Figure S2.** Dissolved dimethylsulfoniopropionate (DMSP) concentrations as a function of time (day) (A, D) in the predator (*K. veneficum* alone) and (B, E) prey (*A. carterae* and *Teleaulax* sp. alone) controls, and in treatments involving incubation of (C) *K. veneficum* with *A. carterae* (*Kv−Ac*) and (F) *K. veneficum* with *Teleaulax* sp. (*Kv−Te*). Different symbols represent different pseudo-replicate experiments, and open and filled symbols indicate the control and experimental bottles, respectively.

**
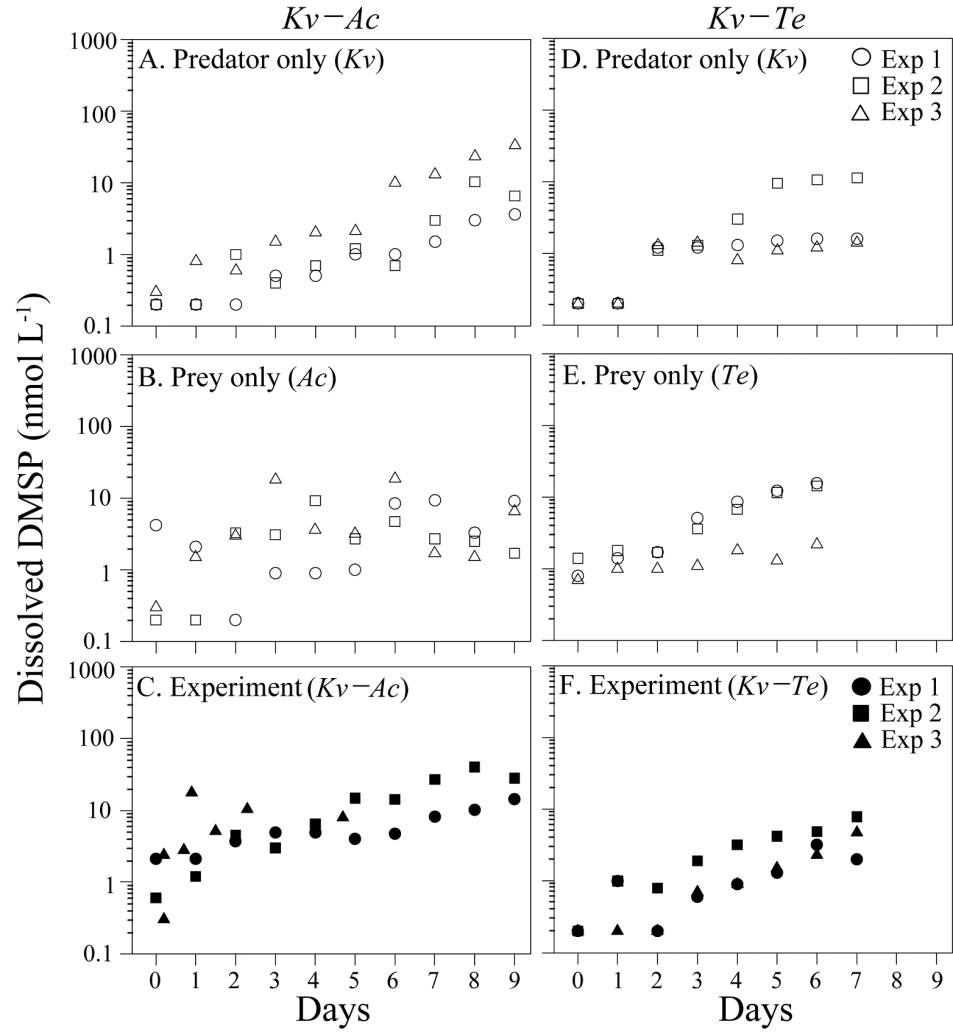
**

**Figure S3.** Relationships between the amount of DMS produced and the amount of DMSP grazed at each dilution level in four dilution experiments in which *K. veneficum* fed on *A. carterae.* The vertical error bars for net DMS production (y-axis) and the horizontal error bars for the grazing rate of DMSP (x-axis) indicate the standard deviations from the mean of replicate measurements. The slope represents the daily DMS production (nmol L^−1^ d^−1^) per grazed DMSP (nmol L^−1^ d^−1^).

**
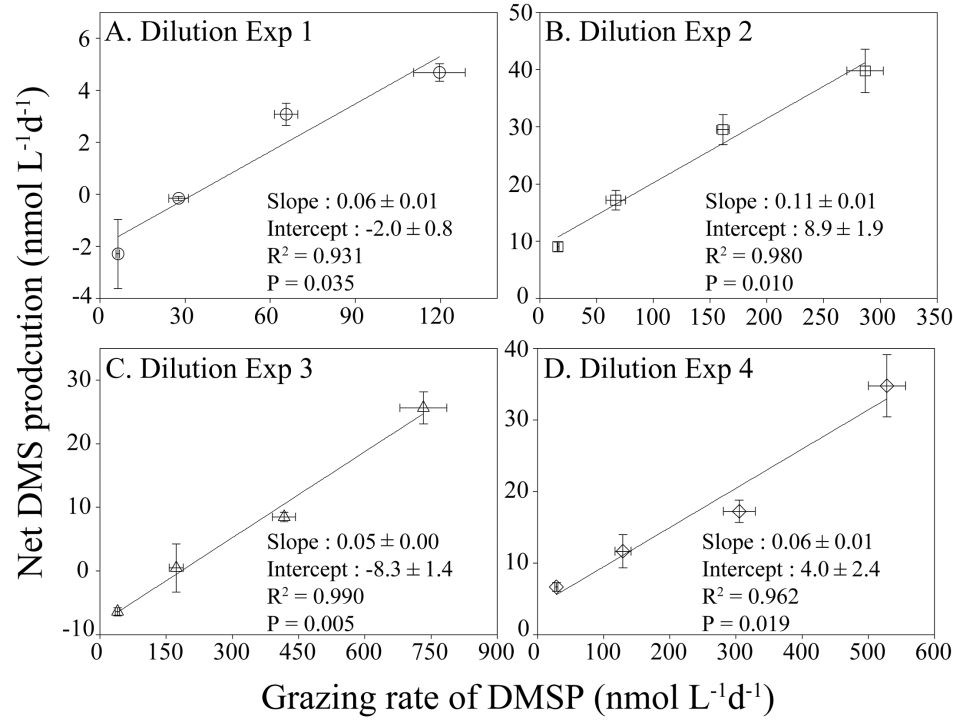
**

**Table S1.** Specific growth rates (d^−1^) of *K. veneficum* and *A. carterae* in controls, and in experimental treatments involving incubation of *K. veneficum* with *A. carterae* (*Kv−Ac*), and ingestion rates (IR, pg C predator^−1^ d^−1^) of *K. veneficum* in experimental treatments. Values in parenthesis indicate errors.

| Day | *K. veneficum* (*Kv*–*Ac*) | | | | | | | | |
| --- | --- | --- | --- | --- | --- | --- | --- | --- | --- |
|  | Experiment 1 | | | Experiment 2 | | | Experiment 3 | | |
|  | Growth rate | | IR | Growth rate | | IR | Growth rate | | IR |
|  | Control | Experiment |  | Control | Experiment |  | Control | Experiment |  |
| 1 | 0.16 (0.05) | 0.20 (0.00) | 88 (2) | 0.42 (0.03) | 0.53 (0.01) | 74 (7) | 0.45 (0.03) | 0.63 (0.011) | 0 |
| 2 | 0.61 (0.04) | 0.78 (0.08) | 6 (10) | 0.69 (0.02) | 0.63 (0.01) | 0 | 0.04 (0.03) | 0.29 (0.14) | 58 (16) |
| 3 | 0.58 (0.04) | 0.58 (0.10) | 15 (6) | 0.71 (0.06) | 0.54 (0.02) | 14 (0) | 1.14 (0.04) | 0.76 (0.09) | 5 (1) |
| 4 | 0.66 (0.01) | 0.66 (0.08) | 50 (5) | 0.72 (0.06) | 0.72 (0.07) | 23 (1) | 0.87 (0.01) | 0.59 (0.01) | 13 (1) |
| 5 | 0.52 (0.03) | 0.67 (0.06) | 12 (2) | 0.79 (0.01) | 0.59 (0.03) | 2 (2) | 0.66 (0.01) | 0.66 (0.07) | 13 (0) |
| 6 | 0.60 (0.03) | 0.38 (0.03) | 27 (1) | 0.48 (0.02) | 0.47 (0.04) | 20 (2) | 0.55 (0.01) | 0.57 (0.06) | 1 (1) |
| 7 | 0.23 (0.02) | 0.53 (0.07) | 32 (1) | 0.44 (0.04) | 0.49 (0.03) | 16 (1) | 0.42 (0.05) | 0.41 (0.04) | 16 (1) |
| 8 | 0.37 (0.01) | 0.46 (0.03) | 7 (1) | 0.03 (0.08) | 0.31 (0.04) | 10 (1) | 0.21 (0.04) | 0.39 (0.04) | 13 (1) |
| 9 | 0.20 (0.02) | 0.22 (0.04) | 1 (0) | 0.36 (0.01) | 0.35 (0.02) | 1 (0) | 0.42 (0.02) | 0.33 (0.03) | 4 (0) |
|  |  |  |  |  |  |  |  |  |  |
| Day | *A. carterae* (*Kv*–*Ac*) | | | | | |  |  |  |
|  | Experiment 1 | | Experiment 2 | | Experiment 3 | |  |  |  |
|  | Growth rate | | Growth rate | | Growth rate | |  |  |  |
|  | Control | Experiment | Control | Experiment | Control | Experiment |  |  |  |
| 1 | 0.70 (0.04) | 0.41 (0.03) | 0.66 (0.04) | –0.35 (0.13) | 0.30 (0.08) | 0.82 (0.13) |  |  |  |
| 2 | 0.37 (0.03) | 0.37 (0.02) | 0.63 (0.04) | 1.31 (0.45) | 0.61 (0.05) | –0.04 (0.01) |  |  |  |
| 3 | 0.49 (0.01) | 0.48 (0.02) | 0.51 (0.01) | 0.34 (0.04) | 0.44 (0.00) | 0.40 (0.06) |  |  |  |
| 4 | 0.59 (0.05) | 0.21 (0.01) | 0.68 (0.03) | 0.29 (0.04) | 0.65 (0.03) | 0.35 (0.06) |  |  |  |
| 5 | 0.55 (0.03) | 0.32 (0.02) | 0.52 (0.00) | 0.53 (0.06) | 0.58 (0.03) | 0.21 (0.03) |  |  |  |
| 6 | 0.36 (0.02) | –0.16 (0.02) | 0.48 (0.06) | –0.08 (0.01) | 0.52 (0.04) | 0.47 (0.04) |  |  |  |
| 7 | 0.49 (0.01) | –1.20 (0.27) | 0.57 (0.03) | –0.32 (0.07) | 0.43 (0.05) | –0.36 (0.05) |  |  |  |
| 8 | 0.25 (0.02) | –1.44 (0.41) | 0.36 (0.07) | –1.37 (0.23) | 0.69 (0.02) | –0.52 (0.07) |  |  |  |
| 9 | 0.15 (0.04) | –7.05 (1.32) | 0.20 (0.05) | –1.05 (0.15) | 0.09 (0.05) | –1.64 (0.15) |  |  |  |

**Table S2.** Specific growth rates (d^−1^) of *K. veneficum* and *Teleaulax* sp. in controls, and in experimental treatments involving incubation of *K. veneficum* with *Teleaulax* sp. (*Kv−Te*), and ingestion rates (IR, ng C predator^−1^ d^−1^) of *K. veneficum* in experimental treatments. Values in parenthesis indicate errors.

| Day | *K. veneficum* (*Kv*–*Te*) | | | | | | | | |
| --- | --- | --- | --- | --- | --- | --- | --- | --- | --- |
|  | Experiment 1 | | | Experiment 2 | | | Experiment 3 | | |
|  | Growth rate | | IR | Growth rate | | IR | Growth rate | | IR |
|  | Control | Experiment |  | Control | Experiment |  | Control | Experiment |  |
| 1 | 0.02 (0.01) | 0.11 (0.00) | 0 | 0.61 (0.07) | 0.27 (0.08) | 0 | 0.71 (0.01) | 0.71 (0.08) | 0 |
| 2 | 0.69 (0.02) | 0.89 (0.01) | 1 (1) | 0.61 (0.09) | 0.88 (0.01) | 3 (3) | 0.29 (0.03) | 0.58 (0.02) | 1 (1) |
| 3 | 0.44 (0.01) | 0.63 (0.01) | 4 (3) | 0.97 (0.01) | 1.00 (0.02) | 18 (8) | 0.42 (0.10) | 0.51 (0.00) | 0 |
| 4 | 0.62 (0.03) | 0.79 (0.04) | 27 (5) | 0.70 (0.04) | 0.96 (0.02) | 0 | 0.82 (0.02) | 0.77 (0.04) | 28 (6) |
| 5 | 0.71 (0.01) | 0.63 (0.02) | 26 (5) | 0.95 (0.04) | 0.61 (0.11) | 6 (4) | 0.82 (0.11) | 0.45 (0.03) | 1 (1) |
| 6 | 0.69 (0.03) | 0.46 (0.06) | 0 | 0.37 (0.15) | 0.54 (0.03) | 0 | 0.64 (0.02) | 0.79 (0.01) | 5 (3) |
| 7 | 0.29 (0.02) | 0.31 (0.04) | 65 (5) |  |  |  | 0.49 (0.10) | 0.41 (0.01) | 5 (0) |
|  |  |  |  |  |  |  |  |  |  |
|  |  |  |  |  |  |  |  |  |  |
| Day | *Teleaulax* sp. (*Kv*–*Te*) | | | | | |  |  |  |
|  | Experiment 1 | | Experiment 2 | | Experiment 3 | |  |  |  |
|  | Growth rate | | Growth rate | | Growth rate | |  |  |  |
|  | Control | Experiment | Control | Experiment | Control | Experiment |  |  |  |
| 1 | 0.44 (0.04) | 0.64 (0.05) | 0.58 (0.03) | 0.68 (0.05) | 0.34 (0.00) | 0.77 (0.11) |  |  |  |
| 2 | 0.92 (0.06) | 0.91 (0.05) | 0.89 (0.01) | 0.99 (0.13) | 0.53 (0.08) | 0.51 (0.05) |  |  |  |
| 3 | 1.10 (0.05) | 1.08 (0.04) | 0.86 (0.03) | 0.71 (0.10) | 0.07 (0.04) | 0.72 (0.05) |  |  |  |
| 4 | 0.92 (0.05) | 0.68 (0.03) | 0.53 (0.03) | 0.77 (0.07) | 1.46 (0.02) | 0.93 (0.15) |  |  |  |
| 5 | 0.81 (0.03) | 0.55 (0.05) | 0.83 (0.08) | 0.80 (0.05) | 0.40 (0.02) | 0.48 (0.07) |  |  |  |
| 6 | 0.56 (0.01) | 0.74 (0.08) | 0.53 (0.03) | 0.70 (0.05) | 0.95 (0.04) | 0.87 (0.03) |  |  |  |
| 7 | 0.65 (0.00) | 0.07 (0.01) |  |  | 0.58 (0.02) | 0.51 (0.02) |  |  |  |

**Table S3.** Cell volumes (μm^3^) of *K. veneficum*, *Teleaulax* sp. and *A. carterae* in the control (CTL) and experimental (EXP) bottles in one of the replicate experiments shown in Fig. 1 (labeled as EXP 1).

|  | Cell volume (μm^3^) | | | |
| --- | --- | --- | --- | --- |
|  | *Kv*−*Ac* pair | | | |
|  | *Kv*−CTL | *Kv*−EXP | *Ac*−CTL | *Ac*−EXP |
| Mean ± SD^a^ | 739 ± 86 | 789 ± 83 | 532 ± 73 | 524 ± 42 |
| N^b^ | 44 | 44 | 47 | 47 |
|  | *Kv*−*Te* pair | | | |
|  | *Kv*−CTL | *Kv*−EXP | *Te*−CTL | *Te*−EXP |
| Mean ± SD | 706 ± 26 | 787 ± 26 | 90 ± 7 | 87 ± 12 |
| N | 47 | 41 | 55 | 51 |

**a.** One standard deviations from the mean of all measurements.

**b.** Number of measurements.
