# Supplementary figures and images for: Prey-dependent retention of dimethylsulfoniopropionate (DMSP) by mixotrophic dinoflagellates
Source: Environ Microbiol. 2012 Mar;14(3):605–16. doi: 10.1111/j.1462-2920.2011.02600.x (PMC3490370; doi:10.1111/j.1462-2920.2011.02600.x)

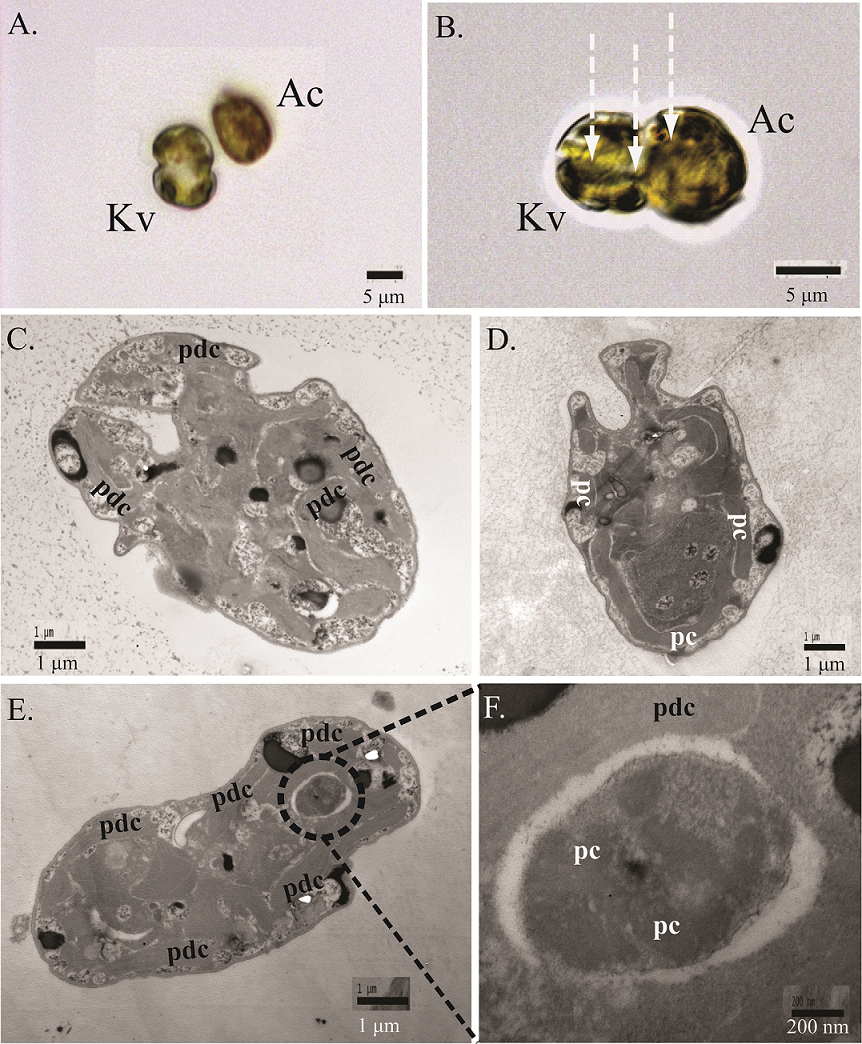

Supplement: Supplementary file 2 — Additional Supporting Information may be found in the online version of this article: Fig. S1. Feeding process of K. veneficum(Kv) on A. carterae (Ac). (A) K.veneficum encountering A. carterae and (B) K.veneficum ingesting the prey cytoplasm (marked as white arrows)through the peduncle and transferring a prey cell to a food vacuoleinside the protoplasm of K. veneficum through the peduncle.Transmission electron microscopy images of (C) K. veneficum,(D) A. carterae, (E) K. veneficum with an ingestedA. carterae cell, and (F) enlargement of the ingested A.carterae cell. Scale bars shown in (A)?(B), (C)?(E) and (F)represent 5 µm, 1 µm and 200 nm respectively. 'pdc' and'pc' in (C)?(F) mean predator and prey chloroplastrespectively. Fig. S2. Relationships between the amount ofDMS produced and the amount of DMSP grazed at each dilution levelin four dilution experiments in which K. veneficum fed onA. carterae. The vertical error bars for net DMS production(y-axis) and the horizontal error bars for the grazing rateof DMSP (x-axis) indicate the standard deviations from themean of replicate measurements. The slope represents the daily DMSproduction (nmol l−1 day−1) pergrazed DMSP (nmol l−1 day−1). Fig. S3. Dissolved dimethylsulfoniopropionate(DMSP) concentrations as a function of time (day) (A, D) in thepredator (K. veneficum alone) and (B, E) prey (A.carterae and Teleaulax sp. alone) controls, and intreatments involving incubation of (C) K. veneficum withA. carterae (Kv?Ac) and (F) K. veneficum withTeleaulax sp. (Kv?Te). Different symbols representdifferent pseudo-replicate experiments, and open and filled symbolsindicate the control and experimental bottles respectively. Table S1. Specific growth rates(day−1) of K. veneficum and A.carterae in controls, and in experimental treatments involvingincubation of K. veneficum with A. carterae(Kv?Ac), and ingestion rates (IR, pg Cpredator−1 day−1) of K.veneficum in experimental treatments. Values in parenthesis indicate errors. Table [file emi0014-0605-SD2.tif]

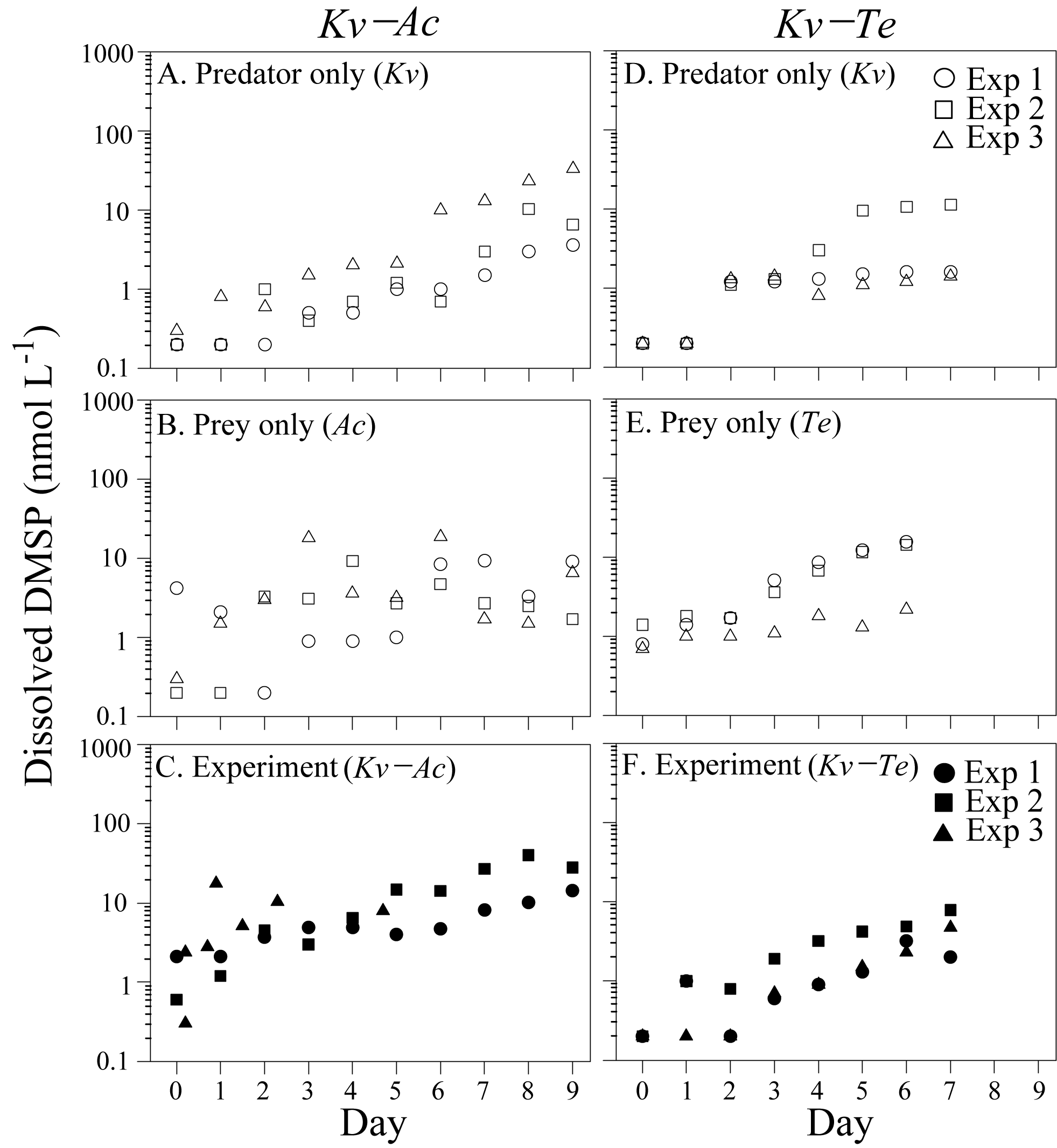

Supplement: Supplementary file 3 — Additional Supporting Information may be found in the online version of this article: Fig. S1. Feeding process of K. veneficum(Kv) on A. carterae (Ac). (A) K.veneficum encountering A. carterae and (B) K.veneficum ingesting the prey cytoplasm (marked as white arrows)through the peduncle and transferring a prey cell to a food vacuoleinside the protoplasm of K. veneficum through the peduncle.Transmission electron microscopy images of (C) K. veneficum,(D) A. carterae, (E) K. veneficum with an ingestedA. carterae cell, and (F) enlargement of the ingested A.carterae cell. Scale bars shown in (A)?(B), (C)?(E) and (F)represent 5 µm, 1 µm and 200 nm respectively. 'pdc' and'pc' in (C)?(F) mean predator and prey chloroplastrespectively. Fig. S2. Relationships between the amount ofDMS produced and the amount of DMSP grazed at each dilution levelin four dilution experiments in which K. veneficum fed onA. carterae. The vertical error bars for net DMS production(y-axis) and the horizontal error bars for the grazing rateof DMSP (x-axis) indicate the standard deviations from themean of replicate measurements. The slope represents the daily DMSproduction (nmol l−1 day−1) pergrazed DMSP (nmol l−1 day−1). Fig. S3. Dissolved dimethylsulfoniopropionate(DMSP) concentrations as a function of time (day) (A, D) in thepredator (K. veneficum alone) and (B, E) prey (A.carterae and Teleaulax sp. alone) controls, and intreatments involving incubation of (C) K. veneficum withA. carterae (Kv?Ac) and (F) K. veneficum withTeleaulax sp. (Kv?Te). Different symbols representdifferent pseudo-replicate experiments, and open and filled symbolsindicate the control and experimental bottles respectively. Table S1. Specific growth rates(day−1) of K. veneficum and A.carterae in controls, and in experimental treatments involvingincubation of K. veneficum with A. carterae(Kv?Ac), and ingestion rates (IR, pg Cpredator−1 day−1) of K.veneficum in experimental treatments. Values in parenthesis indicate errors. Table [file emi0014-0605-SD3.tif]

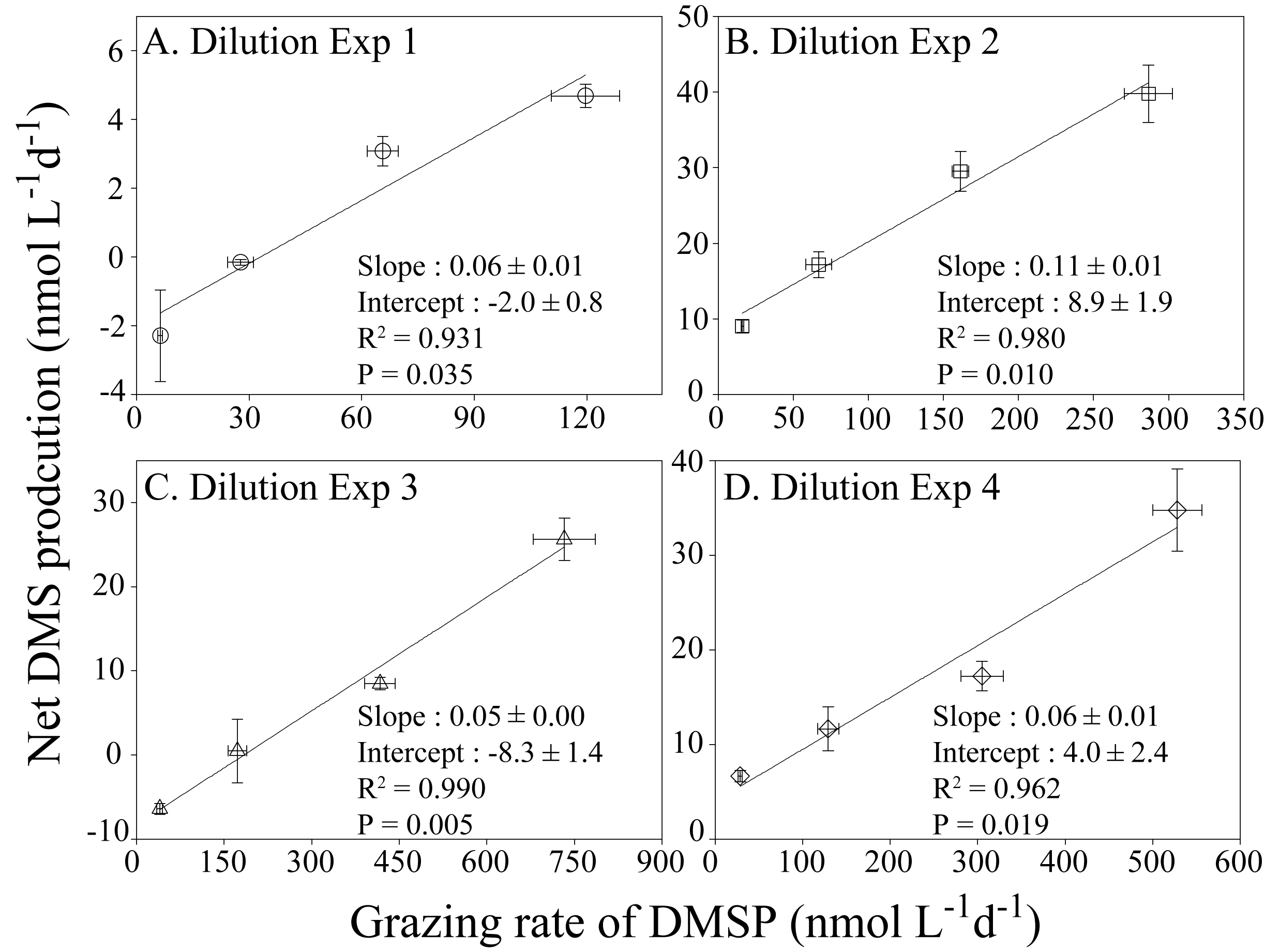

Supplement: Supplementary file 4 — Additional Supporting Information may be found in the online version of this article: Fig. S1. Feeding process of K. veneficum(Kv) on A. carterae (Ac). (A) K.veneficum encountering A. carterae and (B) K.veneficum ingesting the prey cytoplasm (marked as white arrows)through the peduncle and transferring a prey cell to a food vacuoleinside the protoplasm of K. veneficum through the peduncle.Transmission electron microscopy images of (C) K. veneficum,(D) A. carterae, (E) K. veneficum with an ingestedA. carterae cell, and (F) enlargement of the ingested A.carterae cell. Scale bars shown in (A)?(B), (C)?(E) and (F)represent 5 µm, 1 µm and 200 nm respectively. 'pdc' and'pc' in (C)?(F) mean predator and prey chloroplastrespectively. Fig. S2. Relationships between the amount ofDMS produced and the amount of DMSP grazed at each dilution levelin four dilution experiments in which K. veneficum fed onA. carterae. The vertical error bars for net DMS production(y-axis) and the horizontal error bars for the grazing rateof DMSP (x-axis) indicate the standard deviations from themean of replicate measurements. The slope represents the daily DMSproduction (nmol l−1 day−1) pergrazed DMSP (nmol l−1 day−1). Fig. S3. Dissolved dimethylsulfoniopropionate(DMSP) concentrations as a function of time (day) (A, D) in thepredator (K. veneficum alone) and (B, E) prey (A.carterae and Teleaulax sp. alone) controls, and intreatments involving incubation of (C) K. veneficum withA. carterae (Kv?Ac) and (F) K. veneficum withTeleaulax sp. (Kv?Te). Different symbols representdifferent pseudo-replicate experiments, and open and filled symbolsindicate the control and experimental bottles respectively. Table S1. Specific growth rates(day−1) of K. veneficum and A.carterae in controls, and in experimental treatments involvingincubation of K. veneficum with A. carterae(Kv?Ac), and ingestion rates (IR, pg Cpredator−1 day−1) of K.veneficum in experimental treatments. Values in parenthesis indicate errors. Table [file emi0014-0605-SD4.tif]
